# Notes on the Ophthalmological Aspects of Nystagmus

**Published:** 1909-07-10

**Authors:** 


### Ophthalmology.

### NOTES ON THE OPHTHALMOLOGICAL ASPECTS OF NYSTAGMUS.

Nystagmus is a curious, and very often inexplicable, continuous movement of the eyeballs usually towards one side and back to the middle line. It affects both eyes as a rule, but at times it is found in one only. Most frequently it is present at birth or during infancy, and continues throughout life; at other times it is periodic, occurring when the patient is upset or nervously excited: in a large number of cases it is only found when the eyes are directed in, out, up, or down to their extreme limits of rotation. The movements are usually in or out or up and down—that is, the eyes move in the direction of action of the recti muscles; but in rarer cases they are rotatory, and supposed to be produced by the action of the oblique muscles. Sometimes the movements appear to have a purposeful origin, the eyes looking all the time as if trying to see round the corner; this is especially marked should the patient have very defective vision.

The direction of the movement typifies the variety of the nystagmus. If the movements are from side to side or in a vertical direction they are termed nystagmus oscillatorius, horizontalis, or verticalis; if the movement be a rolling one then it is termed nystagmus rotatorius. A combination of these movements is termed accordingly nystagmus mixtus. In general, nystagmus is easy to diagnose; obscure in origin, therefore difficult to account for; and usually impossible to relieve or treat.

The causes of nystagmus are, first and foremost, amblyopia occurring soon after birth or present at birth. Congenital cataract is a not infrequent cause, and so are dense corneal opacities left after an attack of ophthalmia neonatorum, particularly if associated with perforation and consequent anterior polar cataract. Other causes of severe degrees of amblyopia are high refractive errors, albinism, retinitis pigmentosa, or any other degenerated condition of the retina, the result of congenital syphilis.

Nystagmus is also one of the classical symptoms of disseminated sclerosis and it occurs as an evanescent symptom in many cerebral affections. The conjugate deviation of the eyes in brain disease or after a brain injury is frequently nystagmic—that is, not a fixed deviation, but rhythmic in character. Nystagmus occurs as the result of long continuance at certain employments, the most notable of which is mining. The weavers in charge of shuttle machines at times develop nystagmic movements; these, however, are very transient, and rarely if ever persist; they tend to take place only towards one side or the other, and are supposed to be caused by the continual movement of the eyes while watching the moving shuttles.

Nystagmus at times appears in children after they have had an ear syringed, and is then supposed to be occasioned by some irritation of the semi-circular canals; in middle and inner ear disease nystagmus has not infrequently been noted, probably brought about in the same way. Delicate children when cutting teeth have also shown definite transient nystagmus, which has again appeared with the next tooth cut, and at times has preceded convulsions. A combination of nystagmus and head-nodding movements is met with termed spasmus nutans, and for the most part in children the subjects of well-marked rickets. Finally, nystagmus can be produced voluntarily in some patients by the mere act of looking to their extreme limit to one side or the other. Also nystagmus runs in families, and is distinctly hereditary.

Where a certain degree of amblyopia is present soon after birth, there is either a considerable defect in refraction which prevents the child from learning to fix objects, or there is some extensive change in the retina which makes it impossible for a distinct image, though possibly clearly formed, to be perceived by the perceptive elements of the retina. Hence definite fixing of the eyes never takes place, but they travel backwards and forwards in the neighbourhood of the line of fixation; should the retina acquire normal sensitiveness in such cases the nystagmus will gradually disappear. If a child is born blind nystagmus does not occur; the eyes wander aimlessly from side to side, or up and down, but never with the extremely rapid movements of true nystagmus, and the movements traverse a greater area.

A certain degree of vision is necessary before nystagmus can possibly develop; very young children who are unable to fix objects owing to the blurred image formed upon the retina never learn that in a certain position of the eyes they see best, and the actual act of fixation is never acquired. When the eyes are directed towards the object looked at a clearer impression is gained of it than when the eyes are directed elsewhere, but since the clearest image is necessary at the macula and the direct line of vision is obscured, an endeavour is made by extremely rapid movements of the eyeball to place the macula in the best position to receive a defined image. If the retina has never received a clear image no endeavour is made to place the macula in the best position to perceive it.

Miner's nystagmus is probably caused by a combination of the intense darkness and the strained positions in which the men work, lying on their backs with the eyes directed upwards and to one or the other side. This is an acquired form, and curable by the simple expedient of giving up work in a coal mine.

Patients with nystagmus from very early youth do not complain of objects being in motion or dancing, simply because they do not feel the movements of their eyes and have learnt that they can see only while the movements continue. Even then they get only very blurred impressions of objects, so much so that if by an effort they become able temporarily to stop the nystagmic movements compensatory head movements begin. Patients, however, whose nystagmus has developed later in life do complain that objects appear to dance before their eyes since they have learnt to fix with the eyes at rest.

